# Supplementary material for: Are carbon emissions trading and green financial instruments synergistic? -Comprehensive quantitative research based on content analysis
Source: PLoS One. 2024 Mar 7;19(3):e0298601. doi: 10.1371/journal.pone.0298601 (PMC10919601; doi:10.1371/journal.pone.0298601)
Supplement: S1 Data — (ZIP) [file pone.0298601.s001.zip › Data/地级市Z_F工作报告.zip!/║┌┴·╜¡/╝a╬≈╩╨/└┤╫╘╣1⁄2╓┌║┼í╛┬φ┐╦╩2╛▌═°í┐_2019.docx]

各位代表：

现在，我代表市人民政府，向大会报告工作，请予审议。

一、2018年工作回顾

过去的一年，我们以习近平新时代中国特色社会主义思想为指导，在省委、省政府和市委的正确领导下，全面贯彻落实党的十九大精神和习近平总书记在深入推进东北振兴座谈会上的重要讲话精神以及考察黑龙江的重要指示精神，与全市人民一道，迎难而上，砥砺前行，经济社会发展取得新进步，转型发展迈出新步伐。

经济运行稳中向好。预计地区生产总值实现530亿元，增长4%左右。工业质效同升，规模以上工业增加值、主营业务收入、利润分别增长4.5%、15%和6%。经济活力增强，非公经济占全市经济总量的50%以上，电子商务交易额增长30%，快递业务量增长25.6%，银行业金融机构贷款余额增长6.1%。城乡居民人均可支配收入均增长6.5%。

转型发展扎实推进。把产业项目、招商引资、对上争取作为牵动转型发展的重要抓手，全年开复工投资5000万元以上重点产业项目98个，完成投资40.7亿元，中汇石墨、钲祥珈旺豆制品等21个项目竣工投产。全年签约千万元以上项目70个，当年开工建设50个，实际到位资金42亿元，国投公司、哈工大机器人集团等中国500强和行业领军企业入驻鸡西。市本级对上争取资金60亿元，增长9%，争取政府债券资金21.1亿元。加快建设“一都五基地”，天和百万吨焦化、贝特瑞车用负极材料、30万吨玉米燃料乙醇、珍宝岛药业二期等一批项目如期推进，“四大主导产业”产值占工业比重75%以上。旅游经济、口岸经济繁荣活跃，预计旅游业总收入增长11.8%，外贸进出口总额增长10%。

现代农业稳步发展。粮食再获丰收，预计产量68.8亿斤。坚持面向市场调结构，绿色食品种植面积575万亩，鸭稻、蟹稻等发展到1.7万亩，特色经济规模不断扩大，食用菌发展到8千万袋。坚持推进农产品加工转化，开工建设食品产业项目29个，市级以上龙头企业发展到111户。坚持线上线下融合营销，线上销售额2.2亿元，增长61.7%；组建绿色食品产业联盟，在上海、重庆等大中城市建立营销网点195个。新型农业经营主体发展到8949个，增长13%。完成“大棚房”清理整治阶段性任务。农村土地确权颁证率96.7%，48个试点村全部完成改革任务。

改革创新不断深化。坚持以改革打破体制机制束缚，供给侧结构性改革、放管服改革、商事制度改革和国家、省试点改革统筹推进，整治关闭地方煤矿67处；进驻市政务服务中心的187项服务事项，有145项实现“最多跑一次”；市本级政务服务事项流程再造压减审批环节1068个、审批要件540个。国有土地、矿业权、产权项目全部实现电子化交易。坚持以创新驱动产业升级，牵头组建全省北药产业技术创新战略联盟，成立乐新(省级)石墨烯研究院，建立中乌碳晶体重点联合实验室。与东北农大、省农科院建立市院合作关系。全年新认定国家高新技术企业2户，累计达到13户，预计高新技术产业产值实现80亿元，增长10%。

城乡建设推进有力。基础设施建设取得重大进展，牡佳客专(鸡西段)顺利实施，国道丹阿公路(鸡穆界至鸡西段)开工建设，改造农村公路138.7公里。矿区“三供一业”改造一期工程全面完工，二期工程顺利推进。中燃、英联燃气永久场站基本建成，静脉产业园垃圾焚烧发电厂投入运行。城区功能不断提升，维修改造中心区、中心镇道路80条，打造精品示范路19条，改扩建停车场24处，实施绿化亮化工程78处。开展拆违建、治违停、疏拥堵等专项行动，商业区、学校周边环境秩序得到有效整治。污染防治取得阶段性成效，全面落实河(湖)长制，开展清河湖和取缔非法自备井行动，强力推进秸秆禁烧，切实整治工业点源污染和畜禽粪便、农药化肥等农业面源污染。城市集中式饮用水水源地水质达标率100%，市区环境空气质量优良天数326天，比率达到89.8%，同比提升5.5个百分点，完成造林绿化2.1万亩，治理水土流失9万亩。

民生福祉持续改善。全市财政用于民生支出132.9亿元，占一般公共预算支出82.4%。8件民生实事有序推进，1.3万户棚户区改造任务全部开工，89栋老旧住宅小区改造全部完成，新增农村太阳能卫浴一体化用户6000户，改造建设城市水冲公厕65座。百姓关注的回迁安置等民生难题基本化解，累计安置滞留回迁居民4202户，安置率94.5%。723户、1525人实现精准脱贫，贫困村全部脱贫出列。发放各类救助金4.4亿元，惠及群众24万人次。城镇新增就业3.9万人，登记失业率控制在省规定以内。

社会事业全面进步。我市2所学校被认定为全国校长、教师培训基地，39所学校被评为国家和省级特色学校。33家公立医院和公共卫生专业机构药品实现零差率销售；组建家庭医生团队360个，重点人群签约服务31.8万人；15个乡镇卫生院被评为“国家级群众满意乡镇卫生院”。建成鸡西版画馆，成功承办第二届中俄友谊兴凯湖国际马拉松赛、兴凯湖冰雪汽车拉力赛、南岛湖国际冰钓大赛。及时整改中央环保督察、省委巡视“回头看”等反馈意见，361件人大代表、政协委员议案、建议和提案得到认真办理。安全生产形势保持稳定，信访问题得到有效化解，社会治安不断好转，人民群众安全感明显增强。

各位代表，过去一年的成绩来之不易，这是以习近平同志为核心的党中央坚强领导、科学指引的结果，是省委、省政府正确领导、关怀厚爱的结果，是市委总揽全局、科学决策的结果，是市人大市政协有效监督、鼎力支持的结果，是龙煤、农垦、森工等中省直企业携手发展、合作共建的结果，是全市人民同舟共济、团结拼搏的结果。在此，我代表市政府向全市广大干部群众，向所有关心、支持鸡西发展的同志们、朋友们，表示衷心的感谢，并致以崇高的敬意！

在看到成绩的同时，我们也认识到工作中还存在不足和问题：经济稳中趋缓，总体下行仍是主要矛盾；新旧动能转换不快，石墨、绿色食品、生物医药等产业总量不大，支撑力不强；立市、立县(市)区的大项目少，科技型、财源型项目不多；财政收入出现下滑，民生领域还有短板，安全生产压力较大；发展环境有待优化，部分政府工作人员思想不够解放，担当意识不强，不作为、慢作为问题依然存在，等等。对此，我们将坚持问题导向，在今后工作中认真加以解决。

二、2019年工作安排

2019年，是新中国成立70周年，是全面建成小康社会的关键之年。新的一年，既面临国家新一轮支持东北振兴的重大机遇，也面临新旧动能加速转换、新老产业交替发展的重要关口。机遇与挑战同在，压力与动力并存，我们将坚决扛起发展责任，以稳中求进、加大争取、务实苦干为主旋律，以奋发有为的精神状态，以超常规的工作举措，心无旁骛抓发展，久久为功抓落实，不断开创鸡西转型发展新局面。

政府工作的总体要求是：以习近平新时代中国特色社会主义思想为指导，全面贯彻党的十九大和中央经济工作会议精神，聚焦贯彻落实习近平总书记在庆祝改革开放40周年大会上的重要讲话精神，以及在深入推进东北振兴座谈会上的重要讲话精神和考察黑龙江的重要指示精神，按照省、市委经济工作会议部署，坚持稳中求进工作总基调，坚持新发展理念，坚持推进高质量发展，坚持以供给侧结构性改革为主线，坚持深化市场化改革、扩大高水平开放，落实“六稳”要求，围绕突出“一条主线”、叫响“两张名片”、建设“一都五基地”发展战略，大上项目兴产业、创新驱动强实体、改革开放增动力、办好实事惠民生、安全稳定促和谐，全力打造鸡西转型发展升级版，创造全市人民更加幸福美好生活，以优异成绩庆祝新中国成立70周年。

全市经济社会发展的主要预期目标：地区生产总值增长6%；规模以上工业增加值增长6%；固定资产投资增长5%左右；一般公共预算收入增长3%以上；社会消费品零售总额增长8%；外贸进出口总额增长8%；城乡居民人均可支配收入均增长6%。重点做好以下六个方面工作。

(一)聚焦工业强市，扩张升级实体经济

全力开展招商引资。把招商引资作为经济工作的生命线，把握投资导向、跟进投资动向、瞄准前沿方向，谋划储备一批成熟度高、发展前景好的招商项目。树立“舍得”理念，坚持全领域、全要素、全产业链招商，综合运用领导带队招商、以商招商、商会招商、专业社会组织招商、在外鸡西人招商，提高招商引资成效。建立“专班+园区”、“目标+考核”招商引资新机制，设置招商台账，对重大项目实行“保姆式”跟踪服务，提高项目落地实效。把对上争取作为破解发展难题的重要抓手，抓住新一轮东北振兴等机遇，认真研究国家、省扶持方向，以政策、项目、资金为重点，争上级、抢同级、拼社会资本，力争普惠政策全部到位，特殊政策专享到位，竞争政策充分到位。全年对上争取资金增长10%以上。

全力推进产业项目建设。深入实施“重点产业项目建设三年行动”计划，全年开复工投资5000万元以上产业项目95个，当年计划投资83亿元。围绕立市立县(市)区上项目。重点推进30万吨玉米燃料乙醇、哈工石墨3万吨碳基材料等7个投资5亿元以上大项目。围绕主导产业上项目。启动实施龙鑫碳素石墨深加工、三聚绿源生物质综合利用等50个项目。围绕新经济新业态上项目。开工建设首创生态康养、鸡冠跨境电子商务等一批“四新经济”项目。围绕在建项目抓投产。唯大石墨烯产业园一期、同方卓信龙麦啤酒等25个项目实现达产达效。围绕现有企业增效益。推动大唐、青啤、绿都、浩市、珍宝岛等企业，挖潜增效，扭亏增盈，进一步植入资本、技改等要素，推动企业扩张规模、升级发展。

全力做大重点产业。围绕“两张名片”、“一都五基地”建设，制定完善石墨、食品、医药等重点产业发展规划。规模扩张石墨产业。坚持资源开发与精深加工一体化，整合石墨矿权，优化选矿工艺。推进石墨提纯基地建设，尽快形成10万吨高纯石墨产能，逐步延伸打造10万吨负极材料生产基地。鼓励贝特瑞、唯大、普莱德、昌隆石墨等企业发展下游产品，开发终端产品，谋划全产业链产品，推动超高功率石墨电极、超级活性炭、石墨烯制品等精深加工项目建设。依托国家石墨产品质量监督检验中心，加强石墨产业标准制定；推动全国鳞片石墨分技术委员会落户鸡西，提升“中国石墨之都”的话语权和影响力。延伸发展煤炭产业。加快地方煤矿扩储改造，释放优质产能。支持关闭煤矿转型转产，推动洗煤产业转型升级。依托天和焦化、北方焦化等企业，积极推进煤焦油深加工、焦炉煤气制LNG联产、煤层气综合开发利用等项目。培育壮大医药产业。推动珍宝岛药业二期尽快投产，启动三期工程及直接口服中药饮片项目建设，争取珍宝岛医药贸易结算中心落户鸡西。推进乌苏里江制药、野宝药业与上海九州通、肇庆星湖制药等企业合作，扩大产能，拓展市场。增强梨树北药和山产品大市场辐射带动能力。深度开发食品产业。依托现有企业，延伸玉米、水稻等传统加工产业链，提升红小豆、紫苏、蜂产品等特色加工价值链，加快益海嘉里粮油综合加工二期、紫苏油精深加工等项目建设，推动食品产业多元发展、规模发展、深度发展。

全力做好要素保障。突出科技引领。深化市院合作，建设省科学院鸡西石墨分院，筹建唯大石墨烯院士工作站、刺五加保健及药用工程技术中心，支持中乌碳晶体重点联合实验室等申报省级创新基地。新建省级孵化器2家。实施新一轮科技型企业三年行动计划，新增入库科技型中小企业20户。提升园区承载力。坚持对上争一块、招商引一块、财政投一块，多渠道筹措资金，加强园区污水处理、电力、道路、排水等基础设施建设。推进园区安评、环评等统一审批，实现领办代办一门服务，各类审批一次办结，降低入园企业要素成本和制度性交易成本。强化金融支持。充分利用省级七大投资公司平台，着手壮大我市国有资产经营公司规模实力，搭建产业融资服务平台。推进金惠担保公司走向市场，发挥为中小企业融资担保作用。用好多层次资本市场，筛选重点企业“一对一”辅导，支持符合条件的企业挂牌、上市。加强银企对接，鼓励金融机构增加信贷投放。建设人才高地。全面落实人才优惠政策，柔性引进、刚性培养科技领军人才、企业管理人才、技术创新人才和“鸡西工匠”，构建具有较强竞争力的区域性人才高地。

(二)聚焦乡村振兴，提质升级现代农业

稳定粮食生产。坚持农业农村优先发展，落实粮食安全市长责任制，稳步提升粮食产能。坚持“藏粮于地，藏粮于技”，加强与东北农大在种养业、农产品加工等领域的合作共建，加快农业科技研发和成果转化，农业科技贡献率达到68%。推进虎林灌区等重点水利工程建设，实施测土配方施肥等农业新技术，开展轮作休耕，加强黑土地保护，提高农业综合生产能力。

调整优化结构。坚持走特色路。围绕“一乡一业”、“一村一品”，积极发展瓜果蔬菜、食用菌等设施农业，杂粮、杂豆等高效作物，北药、蜂业等林下经济，大白鱼、河蟹等水产养殖。全年种植蔬菜7.6万亩、北药7.5万亩、食用菌1.2亿袋。坚持打绿色牌。农业“三减”示范基地达到125个，“互联网+农业”高标准示范基地达到22万亩，绿色食品种植基地面积600万亩。坚持用典型带。培养选树农民创业典型，发挥示范引领作用，使广大农民干有方向、赶有目标、奔有劲头。

推进融合发展。加强农业生产、加工、仓储、物流、营销、服务等各环节相互融合。大力发展农副产品精深加工业，通过“接二连三”进一步拉长一产链条，提高附加值。培育发展农村电商、农事体验、乡村旅游、休闲农业等新业态新模式，活跃农村经济。实施品牌战略，集中打造“兴凯湖优品”区域公用品牌，积极发展线上线下营销，以品牌营销力倒逼高质量农产品供给力。培育壮大新型经营主体，建立小农户和现代农业发展有机衔接机制，把家庭农场、农民合作社等引入现代农业发展大格局。

建设美丽乡村。实施农村人居环境整治三年行动，加快“四好”公路建设，科学布局建设乡镇污水处理厂、农村垃圾转运站，继续推进农村厕所革命，加强秸秆综合利用，大力开展植树造林。新增农村太阳能卫浴一体化用户6000户、室外公厕30个，绿化村屯108个。推进精准扶贫，农村建档立卡贫困人口全部脱贫，巩固脱贫成果，坚决打赢脱贫攻坚战。大力倡导乡风文明，开展文明村镇、星级文明户创建活动，弘扬时代新风貌，建设既有现代文明，又有田园风光，既望得见山水，又留得住乡情的美丽乡村。

(三)聚焦重点领域，提档升级现代服务业

大力发展旅游业。整合域内旅游资源，串联主要景区景点，规划设计精品线路，集中打造“谜一样的乌苏里江，海一样的兴凯湖，美丽鸡西——行走不一样的江湖”品牌。以兴凯湖、乌苏里江为核心，加强与首旅、泛华深度合作，积极引进战略投资者，推进兴凯湖生态功能区保护和虎林国家全域旅游示范区建设，加强与龙江东部旅游产业联盟城市的集成营销，提升两大核心景区影响力。注重差异化和市场化导向，做精革命文化、生态度假、跨境康养、异国风情等主题游，充分挖掘和传承肃慎、抗联、矿山、北大荒、知青等文化，讲好鸡西故事，推出鸡西篇章，提升旅游发展内涵。瞄准温泉康养、滑雪冰钓等特色项目，发展冬季游，培育做大冰雪经济。推动旅游与文化、体育等融合发展，把中俄跨境马拉松赛、冰雪汽车拉力赛、兴凯湖摄影展、南岛湖国际冰钓大赛打造成全国知名的精品赛事。强化旅游能力提升和市场监管，规范景区管理，优化服务质量，树立鸡西旅游新形象。

加快发展现代服务业。科学规划物流产业。坚持全市“一盘棋”，统一布局园区物流、专业市场物流、产业集群物流、多式联运物流、同城配送物流，加快泛华货运枢纽、华孚冷链物流等项目建设，打造龙江东南部物流中心。推动电子商务等“四新经济”扩量发展。落实支持政策，培育壮大电商经营主体，提升专业策划营销能力，全年电子商务交易额增长25%以上。发挥密山跨境电商产业园作用，围绕农产品输出和跨境贸易，完善综合服务配套体系，培育一批移动终端电商、社区电商、微商，构建跨境电子商务生态圈。积极发展金融、会展、检验检测和咨询认证等生产性服务业。

改造提升传统服务业。坚持以特色带人气、促消费，优化市域商业网点布局，引进知名品牌，丰富商业业态，活跃商业氛围。建设康乐、康新、园林等一批特色街区；引导万达广场、大商新玛特等创新商业模式，加强社区超市、连锁便利店等网点建设，促进老城区商圈转型升级，布局建设鸡冠新区商圈，打造“5分钟生活圈”；积极发展广益城商贸、城子河金三角建材装饰、梨树北药和山产品等专业市场，构建辐射龙江东南部、连接国内外的区域性综合市场，带动人流、物流、资金流、信息流，促进消费。积极培育养老、健康等消费热点，发展大健康产业。

(四)聚焦改革开放，优化升级营商环境

深化改革添动力。持续推进“放管服”改革，确保放得下、接得住、运行好。继续深化“多证合一”改革，推行“35+N”模式和“不见面”审批，实行“网上办、一次办、帮着办”，推进市场主体登记全程电子化。深入推进电力交易、市场化配置资源、公共资源交易电子化等改革。加强教育、医疗、文化等社会改革，推动养老、安全生产等公共服务领域改革，建立起与市场经济相适应的管理体制和运行机制。

扩大开放增活力。对接省“一窗四区”总体布局，主动融入“一带一路”建设。促进对俄开放升级。加强与俄沟通协调，增强口岸通关能力。围绕打造特色口岸，发展俄气俄木俄粮等进口加工，推进对俄开放由出口主导向进口主导、由商贸为主向加工服务为主转变。深化与肇庆对口合作。坚持产业互补、优势共享，加强与肇庆在石墨新材料、农产品加工贸易、北药开发、旅游康养等方面合作，积极推进工业园区共建。加强与农垦、森工、龙煤域内共建。统筹资源、项目和产业，在基础设施、农产品加工、旅游开发、煤层气利用等方面深度合作，共享资源、共建项目、共担责任，打造多赢发展格局。

激活民营强实力。落实支持民营经济发展的各项政策措施，鼓励支持民营资本进入更多领域。加强涉企服务，进一步减税降费，减轻企业负担。研究设立企业信贷风险补偿金、转贷周转金、政府产业引导基金，加强银企对接，解决企业融资难、融资贵问题。建立“亲清”政商关系，真诚坦荡与企业家交朋友，坚持公事不谋私，私事不涉公，不夹带私货，不搞利益交换。制定保护民营经济合法权益办法，依法保护企业家财产权和创新收益，提振民营企业发展信心。以“办事不求人”为目标，开展营商环境评价，加大投诉举报受理办理力度，挂牌督办一批破坏营商环境、新官不理旧账、政府失信违诺等典型案件，营造公平、法治、诚信、重商的良好营商环境。

(五)聚焦宜居宜业，拓展升级城市功能品位

完善城市功能。加快牡佳客专(鸡西段)建设，争取建成兴凯湖外环公路，推进兴凯湖机场改扩建。加强城区“三供一业”、城市排水等基础设施建设。启动餐厨垃圾、污泥处理项目，探索形成高效顺畅的生活垃圾分类系统。实施桥东农贸市场、运输市场等流动、临时性市场异地搬迁，集中建立功能配套、管理规范的综合市场。有序推进市区物流快递公司向物流园区集中。

提升城市品质。实施“添绿”工程。推进主要街路和游园广场绿化，用绿色装点城市。实施“扮靓”工程。做好中心大街等沿街楼体、标志性建筑亮化，鼓励引导企事业单位对文化广场周边高层、新区高层进行亮化，打造特色靓丽的城市天际线。实施“净化”工程。集中清理开放小区、背街巷道、城市出入口等环境卫生，提高保洁水平，提升城市形象品位。

加强生态建设。严格执行生态保护红线制度，全面推进中央、省环保督察和“回头看”反馈问题整改，打赢蓝天保卫战，强化秸秆禁烧，集中整治燃煤小锅炉、老旧车辆和柴油货车污染，减少空气污染排放，让百姓享有更多蓝天白云；打赢碧水攻坚战，深入实施河(湖)长制，坚持一河(湖)一策，实施最严格的水资源管理制度，综合治理穆棱河、兴凯湖，保护好乌苏里江，让江河湖泊的水更清澈；打赢净土持久战，推动绿色矿山建设，加强地质灾害治理，整治土地29.6万亩，退耕还湿1.4万亩，加强城区裸露地治理，还群众一方净土。

提高管理水平。发挥城市管理联席会议作用，统筹各方力量，形成齐抓共管、共建共享的城市管理新机制。加强智慧管理。建立“互联网+智慧城市+数字城管”公共信息平台，加快数字管理指挥中心建设，整合城市管理电话服务平台，实现城市规划、建设、管理、维护全程数字化。突出精细管理。完善市政设施、环境卫生、城市执法等管理标准，推动行业管理全覆盖。加强网格管理，过程管控，细节监督，实现行业管理全过程。推行重点街路、主要商区24小时保洁，365天定点值守，力争综合管理全天候。坚持依法管理。严打黑车等非法营运，深挖“保护伞”，整治客运市场乱象。严治主要街路、商业网点、校园医院周边等交通拥堵。严惩违法占道经营、私搭乱建、违章停放等行为。严管小区物业，将物业管理办法上升为地方法规，推进国有物业公司改革，加强物业市场监管，建立物业企业“黑名单”制度和惩戒退出机制，切实提高物业服务水平。围绕创建全国文明城市，大力倡导文明之风，营造文明城市人人建、违规行为人人管、文明成果人人享的良好氛围。

(六)聚焦民生改善，持续升级百姓福祉

办好10件民生实事。(1)开工建设麻山区引水工程，解决1.5万人饮水难问题。(2)维修改造7个老旧小区45栋楼房，改善居住环境。(3)继续推进国道丹阿公路(鸡穆界至鸡西段)建设。(4)实施朝阳公铁立交桥危桥改造工程。(5)维修改造南星街，同步配套建设停车场及交通设施。(6)新建桥北公交枢纽站及万达、腾飞二期等停车场，开放临街企事业单位停车场，科学划线停车，新增、释放停车泊位5000个，着力解决中心区停车难问题。(7)实施城市基础设施改造升级工程，新建改造供热、供水、供气、排水管网管线25.9公里。(8)开工建设鸡冠新区康新公园。(9)推进全市中小学校教室照明改造。(10)实施覆盖城乡的“天网工程”，增强社会治安防控能力。

统筹社会事业发展。实施积极的就业政策。用足用好省就业创业12条扶持政策，提高就业资金使用效能，增加创业担保贷款申请额度，个人从10万元提高到15万元，小微企业从200万元提高到300万元，简化贷款程序，取消政府出资的融资担保机构“反担保”。做好煤矿转岗职工、退役军人、高校毕业生、农民工等重点群体就业创业服务工作，全年城镇新增就业3万人，登记失业率控制在4%以内。优先发展教育事业。加强幼儿园建设，提高初高中教育质量，解决大班额、大校型、择校问题，整治乱办班、乱补课、乱收费。发展特色化职业教育、特殊教育，支持黑龙江工业学院、黑龙江技师学院加快发展。推进健康鸡西建设。完成市医院新址搬迁，支持市中医院等打造特色专科，加强医护人员培训和继续教育，提高基层医疗卫生服务能力，力争“小病不出县乡，大病不出鸡西”；加强职业病防治、地方常见病诊治，完善医联体建设，做实家庭医生签约服务，努力为群众提供全方位全周期的健康保障。深化医院薪酬制度改革试点，建立现代医院管理制度。积极发展文化事业。以社会主义核心价值观为引领，加强城市文化建设，实施文化惠民工程，提高公共文化服务效能。深入开展诚信鸡西建设。加强殡葬管理，倡导文明祭祀，推进移风易俗。完善社会保障体系。健全社会救助机制，织牢“民生保障网”。明晰市区财政体制，保障区级在编和退休人员工资按时足额发放。

推动社会治理创新。持续打好地方煤矿整治关闭攻坚战，从严落实煤矿安全生产监管措施，推动煤矿安全生产形势持续稳定。全力做好非煤矿山、交通运输、消防森防等重点行业以及学校、商场等人员密集场所安全。加强非洲猪瘟疫情防控，强化食品药品公共安全。建立应急管理协调指挥联动机制，提升应急处置能力。加强退役军人服务管理保障体系建设。落实信访属地管理责任，妥善解决群众合理诉求，该解决的必须解决，该治理的坚决治理。强化社会治安综合治理，深入推进扫黑除恶专项斗争，加大禁毒力度，保持对各类违法犯罪活动的高压打击态势。

三、建设人民满意的实干型政府

各位代表，新时代需要新气象新担当新作为，我们将把“实干”作为政府工作最鲜明的底色，坚持讲实话、重实干、定铁规、树良风，忠诚履职，努力建设人民满意的实干型政府。

提高站位忠诚干。树牢“四个意识”，讲政治、守规矩、顾大局，对标定向，恪尽职守，坚决维护以习近平同志为核心的党中央权威和集中统一领导，坚决贯彻落实省委、省政府各项决策部署，在市委的坚强领导下，以实干展现担当，以有为书写忠诚。

解放思想担当干。坚决破除传统思维、观念障碍，对标先进地区，树立开放思维，站在更高的层次，以更宽广的视野、更大的力度推动鸡西转型发展。强化专业思维、专业素养、专业方法，不断提高政府工作专业化水平。增强斗争精神，敢于动真碰硬，为百姓公平正义、为企业提速发展，无畏压力，勇于担当。大兴调查研究之风，切实为基层解难破阻，推动基层务实干事、激情创业。

明确责任高效干。坚持目标导向，细化量化任务，定性定量定人，上紧责任发条，压实责任担子，做到知责、守责、尽责。强化铁律执行，对定下的事情，以雷厉风行、精益求精的作风，以踏石留印、抓铁有痕的力度，努力把各项工作干出速度、干出质量、干出成效。发挥市营商环境建设监督局作用，加大政府督办检查力度，坚决反对形式主义、官僚主义，坚决整治懒政怠政、为官不为、无所作为、整事混事等行为，确保工作执行有力、落实有果。

依法行政规范干。严格遵守宪法法律，认真执行重大行政决策程序规定，深度推行“双随机一公开”，确保政府工作始终在法治轨道上运行。全面落实市人大及其常委会各项决议决定，办好人大代表议案建议和政协提案，自觉接受人大依法监督、政协民主监督以及社会舆论监督。深入开展“七五”普法，推动法治鸡西建设。

筑牢底线廉洁干。加强政府廉政建设，严格遵守廉政纪律，守住底线，不踩红线，不碰高压线。加大对重点领域、关键环节、重要岗位权力运行的管理力度，进一步规范公共资源交易、政府采购等行为。坚持厉行节约，加大政府支出控制力度，严控“三公”经费支出，树立政府清廉节俭良好形象。

各位代表，打造转型发展升级版，是我们笃定前行的方向；重振鸡西雄风，是我们矢志不渝的追求。让我们更加紧密团结在以习近平同志为核心的党中央周围，在省委、省政府和市委的坚强领导下，瞄准方向、真抓实干、埋头苦干、久久为功，为开创鸡西全面振兴、全方位振兴新局面而努力奋斗！
